# Regulatory Experiences with the Use of Multiple Imputation for Missing Data in a Phase 3 Confirmatory Trial

**DOI:** 10.1007/s43441-025-00872-1

**Published:** 2025-10-10

**Authors:** Mouna Sassi-Sayadi, Pierre Verweij, Peter Cornelisse

**Affiliations:** Idorsia Pharmaceuticals Ltd, Hegenheimermattweg 91, Allschwil, 4123 Switzerland

**Keywords:** Missing data, Multiple imputation, MMRM, Regulatory agencies, Sensitivity analyses, Regulatory interactions

## Abstract

The Mixed Model for Repeated Measures (MMRM) is widely used in clinical trials, however, its reliance on the Missing at Random (MAR) assumption and the exclusion of subjects lacking post-baseline data have been points of scrutiny, particularly due to misalignment with the Intent-to-Treat (ITT) principle. This paper presents an application of Multiple Imputation (MI) to address missing data in a hypertension clinical trial and discusses the subsequent interactions with regulatory authorities requesting additional analyses predominantly based on a Missing not at Random (MNAR) assumption. While MNAR-based approaches have been traditionally used for sensitivity analyses, we present an example demonstrating that regulatory agencies are increasingly expecting their integration into primary analyses.

**Supplementary Information:**

The online version contains supplementary material available at 10.1007/s43441-025-00872-1.

## Introduction

The Mixed Model for Repeated Measures (MMRM) is frequently used as a primary analysis method in clinical trials [1]. However, the validity of the underlying Missing at Random (MAR) assumption is often questioned by regulatory authorities, limiting its acceptance. MMRM also excludes subjects without post-baseline data, which is not in line with the ITT principle—a fundamental standard in confirmatory clinical trials.

Over the past decades, various methods have been proposed to handle missing data in clinical studies. Among them, multiple imputation (MI) has become widely adopted, particularly as advances in computing power made it feasible. MI enables statistically robust handling of incomplete datasets, reducing bias compared to ignoring missing data. In practice, it is commonly used in sensitivity analyses [2]. For instance, reference-based or control-based MI under the Missing Not at Random (MNAR) assumption to challenge the main analysis [3, 4], which typically assumes MAR. While MAR assumes missingness can be predicted from observed data, MNAR accounts for scenarios where missingness depends on unobserved factors.

In recent years, use of MI based on MNAR assumptions has increased in primary analyses, particularly in regulatory settings, reflecting growing recognition that the MAR assumption may not always hold.

This paper illustrates the application of MI in sensitivity and, ultimately, primary analyses, drawing from our experience gained during the regulatory review of a new drug for resistant hypertension (RHT). We do not intend to cover all aspects of MI here. This information can be found in Cro et al. [5], Wang et al. [6] or Bell et al. [7] but we highlight a practical application of a combined approach using MI under MAR and MNAR assumptions, considering patient completion status and reasons for missingness in the main analysis. Throughout, focus is on the treatment policy estimand as this was defined as the primary estimand and not questioned during the regulatory review.

## Background

The presented data are from the PRECISION trial, a multicenter, blinded, randomized, parallel-group study with aprocitentan in RHT [8].

**Fig. 1:**
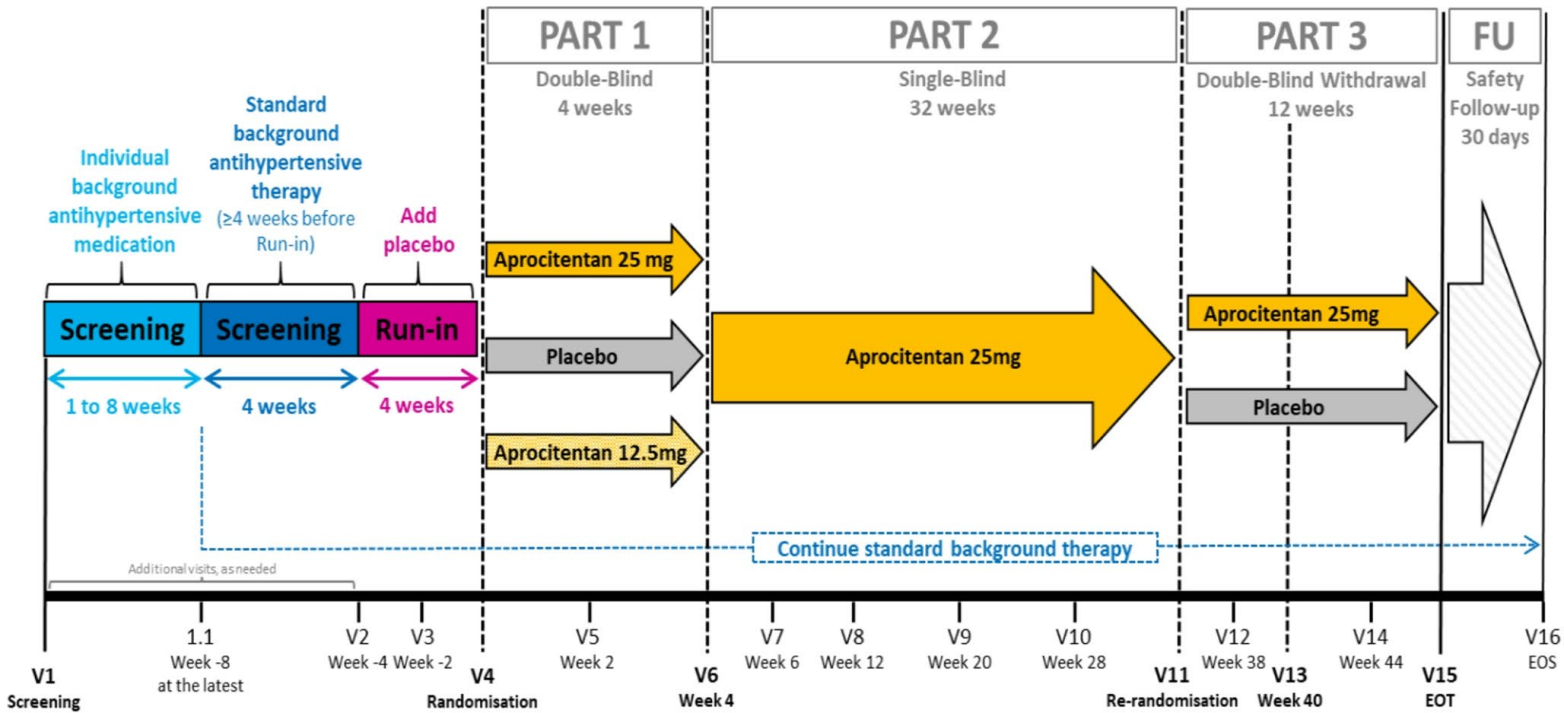
Design of the PRECISION study [17]

The subjects were eligible for randomization if their sitting systolic blood pressure (SiSBP) was 140 mmHg or higher despite taking three antihypertensive drugs, including a diuretic. Before randomization, the subjects’ background medication was switched to a standardized combination of an angiotensin receptor blocker, a calcium channel blocker and a diuretic. The study consisted of three sequential parts: 4-week double-blind, randomized, and placebo-controlled part, where subjects received aprocitentan 12,5 mg, aprocitentan 25 mg, or placebo in a 1:1:1 ratio; 32-week single (patient)-blind part, where all subjects received aprocitentan 25 mg; and 12-week double-blind, randomized, and placebo-controlled withdrawal part, where subjects were re-randomized to aprocitentan 25 mg or placebo in a 1:1 ratio (Fig. 1). The Phase 3 study is registered on ClinicalTrials.gov, NCT03541174.

This study is two studies in one: The double-blind phase aimed to demonstrate the BP-lowering effect of aprocitentan in subjects with RHT (Part 1), while Parts 2 and 3 were intended to evaluate the persistence of this effect over time.

Here, 730 subjects were randomized in double-blind part and 614 subjects were re-randomized in the withdrawal part. The primary and key secondary endpoints were changes in unattended office SBP from baseline to week 4 and from withdrawal baseline to week 40, respectively. Here we focus on the analyses of the primary endpoint. The analyses of the key secondary endpoint is in the supplement section.

## Methods

The pre-specified primary endpoint analysis used an MMRM with factors for treatment group (aprocitentan 12.5 mg, 25 mg and placebo), visit (week 2, week 4), treatment-by-visit interaction, and continuous covariates for baseline SiSBP and baseline by visit interaction. An unstructured covariance matrix accounted for correlations between repeated BP measurements from the same subject. Least Squares (LS) mean differences vs. placebo at Week 4 (along with two-sided 97.5% CIs) were obtained from this model. Aprocitentan 12.5 mg vs. placebo and 25 mg vs. placebo were tested at a two-sided significance level of 0.025. Two types of intercurrent events were defined in this study, premature discontinuation of double blind (DB) treatment and the addition or dose increase of a diuretic (other than the one already present in the standardized background medication) (Table 1). All automated office blood pressure measurements (AOBPM) measurements, irrespective of any intercurrent event(s), were included in the main analysis, leading to a treatment policy strategy estimand in ICH terminology [9]. A hypothetical strategy estimand was also considered, excluding AOBPM data collected after the occurrence of any intercurrent events. The other attributes were the same for both estimands: the treatment was aprocitentan 12.5 or 25 mg, the target population were patients with RHT, the endpoint was change from baseline to week 4 in SBP and the summary measure was the mean change from baseline.

**Table 1:** Overview of intercurrent event

Treatment	Aprocitentan 12.5 mg N = 243	Aprocitentan 25 mg N = 243	Placebo N = 244	Total N = 730
Premature treatment discontinuation	11 (4.5%)	9 (3.7%)	6 (2.5%)	26 (3.6%)
Rescue medication or use of diuretic	9 (3.7%)	16 (6.6%)	4 (1.6%)	29 (4.0%)

In the MMRM-based main analysis, missing data were not imputed but handled under the MAR assumption. The impact of deviations from the MAR assumption underlying the mixed model was investigated in two prespecified control-based multiple imputation procedures (copy-reference (CR) and jump-to-reference (J2R) [3, 4]) assuming MNAR for aprocitentan but MAR for placebo. Multiple imputation of missing values was performed assuming that subjects with missing data in the aprocitentan arms would immediately follow the trajectory of the placebo arm (for J2R) or that treatment effect gradually diminished post-dropout (for CR), conditional on subjects’ data available prior to discontinuation. Technically, this was done as follows: first, any non-monotone missing data (i.e., BP missing at Week 2, but observed at Week 4) were imputed under MAR. Then, imputation was sequential, one visit at a time [10], with a regression model fitted using placebo data to predict outcomes for missing aprocitentan data. The imputation models included as covariates: baseline and the value at week 2 and/or week 4.

A tipping point analysis assessed the severity of deviations from the MAR assumption for overturning the conclusions of the main analysis [11]. In this analysis, missing data were first imputed under MAR (also including treatment group in the imputation model), then the aprocitentan groups were penalized by adding a shift parameter *s* mmHg to each imputed value. This was done for a series of *s* values ranging from 0 to 10 mmHg. Only positive shift parameters were considered since these corresponded to a smaller reduction or larger increase from baseline to Week 4 in SBP. For each shift parameter the multiple data sets were analyzed using Analysis of Covariance for the last time point (which gives the same results at that time point as the MMRM specified for the main analysis).

SAS software version 9.4 was used for all MI analyses using PROC MI as well as an in-house macro for tipping point analyses.

Per common practice, the analysis incorporated visit windows. The window for Week 2 (Day 15) was Day 8–20, whereas the window for Week 4 (Day 29) was Day 21–36. If multiple measurements existed within the same window, the value closest to the target day was used. This approach sometimes led to the ‘creation’ of missing data, for example, when a Week-2 visit occurred in the Week-4 window, thus leading to two candidate Week-4 values (of which only one was chosen).

For the primary analysis, most missing data resulted from visits outside the predefined analysis windows (Table 2). Both week 2 and week 4 data were missing for 3% of the subjects.

**Table 2:** Overview of missingness patterns

Treatment	Aprocitentan 12.5 mg N = 243	Aprocitentan 25 mg N = 243	Placebo N = 244	Total N = 730
Total missingness	20 (8.2%)	12 (4.9%)	20 (8.2%)	52 (7.1%)
*Cause missingness*				
No post baseline visits in study	2 (0.8%)	1 (0.4%)	1 (0.4%)	4 (0.5%)
Visit outside analysis windows ([21;36])	10 (4.1%)	8 (3.3%)	9 (3.7%)	27 (3.7%)
Week 4 visit not performed (other reasons like treatment discontinuation or visit missed)	8 (3.3%)	3 (1.2%)	10 (4.1%)	21 (2.9%)

## Results

### Planned Analyses

In the primary analysis using the treatment policy estimand, both aprocitentan 25 mg and 12.5 mg showed a statistically significant difference versus placebo in the mean SiSBP change from baseline to Week 4 (Table 3). The hypothetical strategy estimand showed similar results (LS mean change vs. placebo: -3.41 mmHg *p*-value = 0.0109 for 12.5 mg, − 3.73 mmHg *p*-value = 0.0054 for 25 mg),

**Table 3:** Overview of the sponsor’s main sensitivity analyses—difference to placebo for changes in SiSBP (mmHg) from baseline to week 4 (uAOBPM): FAS

Analysis	Aprocitentan 12.5 mg		Aprocitentan 25 mg	
	LS Mean	*p*-value	LS Mean	*p*-value
Main analysis	− 3.79	0.0042	− 3.73	0.0046
*Sensitivity analyses*				
J2R	− 3.63	0.0062	− 3.52	0.0076
CR	− 3.58	0.0072	− 3.65	0.0056
*Tipping point*				
Delta = 0 mmHg	− 3.78	0.0041	− 3.74	0.0043
Delta = 2 mmHg	− 3.61	0.0061	− 3.64	0.0054
Delta = 4 mmHg	− 3.45	0.0089	− 3.54	0.0069
Delta = 6 mmHg	− 3.28	0.0129	− 3.44	0.0087
Delta = 8 mmHg	− 3.12	0.0185	− 3.35	0.0111
Delta = 10 mmHg	− 2.95	0.0262	− 3.25	0.0140

CR = copy-reference; FAS = Full analysis set; J2R = jump-to-reference; LS mean = least squares mean; SiSBP = sitting systolic blood pressure; uAOBPM = unattended automated office blood pressure measurement

Sensitivity analyses yielded results consistent with the main analysis, with slightly lower effect sizes but p-values remaining below the significance level of 0.025 (Table 3).

In the tipping point analysis, the statistical significance for aprocitentan 12.5 mg versus placebo was overturned only at a shift parameter of 10 mmHg (*p* = 0.0262). The difference between aprocitentan 25 mg and placebo was still statistically significant (*p* = 0.0140) when using this maximum shift parameter. All analyses summarized in Table 3 were submitted to health authorities as part of the aprocitentan regulatory submission.

### Additional Analyses Requested by Regulatory Authorities

During aprocitentan’s regulatory review, health authorities raised concerns about missing data assumptions for primary endpoint (e.g., the MAR assumption for subjects who discontinued treatment or received rescue medication) and requested additional sensitivity analyses, including retrieved dropouts and return to baseline. They also noted that the main analysis (MMRM) did not fully adhere to the ITT principle. The key requests are summarized in the next section.

### Retrieved Dropouts and Return To Baseline

The first agency requested additional analyses to impute missing data, distinguishing subjects who completed the 4 weeks of DB treatment from who prematurely discontinued. Four categories of subjects were created:

- Completed treatment DB part 1 and had Week 4 data (**A**).
- Did not complete treatment DB part 1 but had Week 4 data (**B**).
- Completed treatment DB part 1, but with missing or out-of-window data at Week 4 (**C**).
- Did not complete treatment DB part 1 and had missing or out-of-window data at Week 4 (**D**).

To address this, imputation was performed using the retrieved dropout methodology [6, 7, 12] rather than imputing missing data based on all available non-missing data, as was done in other sensitivity analyses. Two separate imputations were conducted:

**Table:** 

Missing data in:	Imputed based on:	Methodology
**A.** Subjects who completed treatment DB part 1, but with missing or out-of-window data at Week 4; *n* = 37	**B.** Subjects who completed treatment DB part 1 and having Week 4 data; *n* = 667	MAR
**C.** Subjects who did not complete treatment DB part 1 and had missing or out-of-window data at Week 4; *n* = 15	**D.** Subjects who did not complete treatment DB part 1 but had Week 4 data; *n* = 11 (retrieved dropouts)	MAR

In both cases, multiple imputations were performed under the MAR assumption. In each replication, a pair of imputed data sets (*n* = 704 and *n* = 26) was combined and analyzed using Rubin’s [13] method.

As expected, imputing missing data for 15 subjects (C) using data from only 11 subjects (D) led to computational issues, including the rejection of imputed datasets following the imputation of implausible values. The analysis was based on the accepted datasets which were slightly fewer than the requested number. Excluding the ‘unacceptable’ datasets probably had a minor impact on the results but was inconvenient. This and other issues with data sparsity have also been discussed by Bell et al. [7].

The regulatory agency also requested an additional analysis using return to baseline multiple imputation [6, 14, 15], assuming that the potential was drawn from the same distribution as baseline.

The results of these (Table 4) were consistent with those from the main analyses from the CSR, with LS Mean differences only slightly impacted. and p-values remaining below the significance level of 0.025.

**Table 4:** Overview of additional sensitivity analysis requested by regulatory agencies—difference to placebo for changes in SiSBP (mmHg) from baseline to week 4 (uAOBPM), full analysis set

Analysis	Aprocitentan 12.5 mg		Aprocitentan 25 mg	
	LS Mean	*p*-value	LS Mean	*p*-value
Main analysis for SiSBP	− 3.79	0.0042	− 3.73	0.0046
*Regulatory agency 1*				
Multiple Imputation using retrieved dropout for SiSBP*	− 3.63	0.0107	− 3.43	0.0126
Baseline Multiple Imputation for SiSBP*	− 3.42	0.0117	− 3.83	0.0044
Implement ITT principle by Multiple imputation under MAR (rejected by agency)	− 3.78	0.0043	− 3.70	0.0049
Implement ITT principle by Multiple imputation using retrieved drop out (after discussion with agency)	− **3.79**	**0.0043**	− **3.65**	**0.0054**
*Regulatory agency 2*				
Multiple imputation (1) analysis	− 3.76	0.0044	− 3.63	0.0059
Multiple imputation (2) analysis	− 3.75	0.0045	− 3.61	0.0061
J2R analysis	− 3.63	0.0062	− 3.52	0.0076

The result in bold was eventually included in the Agency’s label

SiSBP = sitting systolic blood pressure; J2R = Jump to reference

*Among the 250 imputed datasets, a few datasets could not be created and were excluded from the analysis due to low subject numbers in category C and D

### The ITT Principle

Later in the review process, the agency commented that the MMRM violated the ITT principle—in that subjects without any post-baseline value were (implicitly) excluded from the analysis.

Since the number of subjects without post-baseline blood pressure data was low (3%), a MAR imputation of all missing data before running the mixed model showed that the results were consistent (LS mean change vs. placebo: − 3.78 mmHg *p*-value = 0.0043 for 12.5 mg, − 3.70 mmHg *p*-value = 0.0049 for 25 mg (Table 4).

However, this proposal based on MAR was not accepted. In response, the regulatory agency issued a follow-up information request proposing a different way of imputation, which was like the retrieved dropouts described above, combining MAR and MNAR imputation to handle missing data for the primary analysis.

**Table:** 

Missing data in:	Imputed based on:	Methodology for imputation
**A**. Subjects who completed treatment DB part 1, but with missing or out-of-window data at Week 4; *n* = 37	**B**. Subjects who completed treatment DB part 1 and having Week 4 data; *n* = 667	Under MAR assumption
**C**. Subjects who did not complete treatment DB part 1 and had missing or out-of-window data at Week 4: *n* = 15	**E**. Other subjects (A, B, D) *n* = 715	Under MNAR assumption J2R

Note that categories A–C are the same as used in the retrieved dropouts. However, category D from the previous analysis was too small (11 subjects) to run MI under MNAR using J2R. Therefore, a J2R using all data as in the pre-specified sensitivity analysis (Category E) was applied and the subset of 26 subjects (11 with data at week 4 and 15 imputed with J2R) were extracted to pursue the analysis. This approach was accepted by the regulatory agency.

The results were close to those of the previously “rejected” analysis: LS mean change versus placebo: − 3.79 mmHg *p*-value = 0.0043 for 12.5 mg, − 3.65 mmHg *p*-value = 0.0054 for 25 mg (Table 4).

Ultimately the regulatory agency incorporated this analysis into the final label document.

A second regulatory agency also highlighted the ITT principle for subjects with missing post-baseline assessments and requested additional sensitivity analyses for the primary endpoint. The proposed multiple imputation analyses based on the reason for missingness were:

**Table:** 

Reason for missing data	Multiple imputation analysis (1)*	Multiple imputation analysis (2)
Treatment discontinuation	J2R	J2R
Rescue medication or addition/dose increase of a diuretic	MAR	J2R
Other	MAR	MAR

*If in multiple imputation analysis (1) a subject has both treatment discontinuation and rescue medication (or addition/dose increase of a diuretic) as a reason for missing data, this subject’s data will be imputed using J2R (conservative approach)

The results of the 2 additional sensitivity analyses are summarized in Table 4. A logical third MI analysis would impute all missing data using J2R, already reported in Table 3 and repeated in Table 4 for completeness.

For the primary endpoint, *p*-values increased slightly as the sensitivity analysis became more conservative. In particular, the results for the multiple imputation (1) and (2) by reasons of missing data fall between MAR and J2R, reinforcing the robustness of the main analysis.

This agency accepted our responses and did not request any additional analysis due to the low number of missing data and consistency with primary results.

In their integrated review [16], the first regulatory agency not only mentioned their main concerns with our prespecified primary analysis using MMRM (i.e., exclusion of subjects with missing post-baseline data and assuming MAR), but also indicated that a retrieved dropout multiple imputation method is usually their preferred approach for handling missing data due to dropout or withdrawal.

However, they acknowledged that the retrieved dropouts were too few to use this approach reliably.

The agency’s final label included an analysis imputing missing data using a combination of 2 approaches:

1. MNAR: Jump-to-reference multiple imputation for subjects who did not complete DB treatment.
2. MAR: Standard multiple imputation for the rest of the missing data.

This method aligns with the ITT principle by including all subjects and ensuring missing data were handled appropriately before analyzing the primary analysis. The results were similar to the prespecified primary analysis as shown in Table 3.

## Discussion and Conclusion

We have described our experience with the regulatory review process following the submission of a new drug for resistant hypertension. While our “n = 1” experience does not define a general regulatory approach to missing data in visit-based studies, we believe it provides useful insights. Simple approaches like complete case analyses and last observation carried forward, have been replaced by MMRM [1], with the MAR assumption often reasonable in controlled trials [2]. We performed MNAR sensitivity analyses using “a-one-size-fits-all approach” (i.e., CR, J2R or tipping point, regardless the reason of missingness), despite only 3% of subjects missing both week 2 and week 4 data.

Most regulatory review requests involved additional sensitivity analyses using MI under MNAR but deviating from the “one-size-fits-all” approach. One agency requested imputing missing Week-4 data for treatment completers using data from other completers, and for non-completers using data from other non-completers, despite the small numbers. Another agency recommended MAR or J2R depending on the reason—treatment discontinuation or diuretic use (both intercurrent events). Given the low missing data rate (3%), these MI analyses yielded results consistent with the main analysis.

The standard MMRM excludes subjects without post-baseline BP data, thus violating the ITT principle. In our study, only 3% lacked post-baseline data, so the issue was not emphasized in the original application. However, both regulatory agencies requested further analysis, where MI proved effective. Performing MI before the main analysis is a simple way to align with ITT. The agencies raised no concerns about estimand choices. The primary estimand followed a treatment policy strategy, including all BP values regardless of intercurrent events (e.g., discontinuation, diuretics, rescue medication). The hypothetical estimand excluded post-event data and yielded similar results.

Regulatory-requested analyses remained consistent with the primary analysis, reflecting the minimal missing data. Discussions with agencies helped refine the MI strategy ultimately accepted for drug labeling.

One agency requested the retrieved dropout method, imputing completers from other completers and non-completers from non-completers, applying MAR and J2R respectively. Another agency selected MI methods based on missing data reasons—J2R for treatment discontinuation, and either MAR or J2R for rescue medication or diuretic use.

Our interactions with regulatory agencies highlight growing emphasis on tailoring MI to completion status or missingness reason. While feasible for short-term endpoints, this becomes complex for long-term endpoints, particularly with limited retrieved dropouts. It was acknowledged by the agency that there were not enough retrieved dropouts to support a reliable retrieved dropout multiple imputation. This underlines once more the importance of collecting all primary endpoint data, including post-treatment discontinuation data.

Selecting the appropriate MI methods requires evaluation of missingness patterns during the trial. We hope our experience further enhances the understanding and application of these concepts.

## Supplementary Information

Below is the link to the electronic supplementary material.

Supplementary Material 1

## Data Availability

No datasets were generated or analysed during the current study.
