# Supplementary material for: Regulatory Experiences with the Use of Multiple Imputation for Missing Data in a Phase 3 Confirmatory Trial
Source: Ther Innov Regul Sci. 2025 Oct 10;60(1):8–14. doi: 10.1007/s43441-025-00872-1 (PMC12753554; doi:10.1007/s43441-025-00872-1)
Supplement: Supplementary file 1 — Supplementary Material 1 [file 43441_2025_872_MOESM1_ESM.docx]

**SUPPLEMENTARY MATERIAL**

**Analyses conducted for the key secondary endpoint**

In the DB-WD part, 614 subjects were re-randomized. For the part 3, a statistically significant difference to placebo was observed for change in SiSBP from DB-WD baseline to Week 40 in the aprocitentan 25 mg group.

An overview of the missingness data pattern for the key secondary endpoint is provided in table S1. For the key secondary analysis, most of the missing data resulted from visits occurring outside the predefined analysis windows.

The same sensitivity analyses were conducted for the key secondary endpoint as for primary endpoint by the sponsor as shown in table S2. For the key secondary endpoint, all were consistent with the main analysis. All p-values were very low (p < 0.0001) and remained far below the significance level of 0.05.

Similar analyses were conducted on the key secondary endpoint in the DB-WD part based on regulatory agency requests. These multiple imputations were performed for the treatment policy estimand.

Retrieved dropout imputation was conducted as follow:

| Missing data in: | Imputed based on: | Methodology |
| --- | --- | --- |
| A. Subjects who completed treatment DB-WD part 3, but with missing data at Week 4; n = 71 | B. Subjects who completed treatment DB part 1 and having Week 4 data; n = 506 | MAR |
| C. Subjects who did not complete treatment DB-WD part 3 and had missing data at Week 4; n = 15 | D. Subjects who did not complete treatment DB-WD part 3 but had Week 4 data; n = 22 (retrieved dropouts) | MAR |

In both cases, multiple imputations were performed under the MAR assumption. In each replication, a pair of imputed data sets (n=578 and n=37) was combined and analyzed using Rubin’s [1987] method.

Ultimately, as discussed for the DB part, the following MI was conducted:

| Missing data in: | Imputed based on: | Methodology for imputation |
| --- | --- | --- |
| A. Subjects who completed treatment DB-WD part 3, but with missing data at Week 4; n = 71 | B. Subjects who completed treatment DB part 1 and having Week 4 data; n = 506 | Under MAR assumption |
| C. Subjects who did not complete treatment DB-WD part 3 and had missing data at Week 4; n = 15 | E. Subjects in all other categories (A,B,D); n=592 | Under MNAR assumption J2R |

The regulatory agency 2 also requested similar analysis according to reasons of missingness in the DB-WD part.

| Reason for missing data | Multiple imputation analysis (1) * | Multiple imputation analysis (2) |
| --- | --- | --- |
| Treatment discontinuation | J2R | J2R |
| Rescue medication or addition/dose increase of a diuretic | MAR | J2R |
| Other | MAR | MAR |

*If in multiple imputation analysis (1) a subject has both treatment discontinuation and rescue medication (or addition/dose increase of a diuretic) as a reason for missing data, this subject’s data will be imputed using J2R (conservative approach).

Table S3 shows all the multiple imputations conducted for the 2 regulatory agencies along with the main analysis as a benchmark.

The results of the multiple imputation analyses for the treatment policy strategy were consistent with those from the main analyses from the CSR, with LS Mean differences only slightly impacted, and p-values remaining below the significance level of 0.05.

**Table S1. Overview of missingness patterns for the key secondary endpoint**

| **Treatment /**  **Cause missingness** | **Aprocitentan 25 mg N=307** | **Placebo N=307** | **Total  N=614** |
| --- | --- | --- | --- |
| Visit outside analysis windows ([20;41]) | 38 (12.4%) | 24 (7.8 %) | 62 (10.1%) |
| No post baseline visits in study | 1 (0.3%) | 2 (0.6 %) | 3 (0.5%) |
| Week 40 visit not performed (other reasons like treatment discontinuation or visit missed) | 7 (2.3 %) | 14 (4.6 %) | 21 (3.1%) |

**Table S2. Overview of the sponsor’s main sensitivity analyses – difference to placebo for changes in SiSBP (mmHg) from DB-WD baseline to Week 40 (uAOBPM): mFAS**

|  | **Aprocitentan 25 mg** | |
| --- | --- | --- |
| **Analysis** | **LS Mean** | **p-value** |
| Main analysis | -5.82 | < 0.0001 |
| Sensitivity analyses |  |  |
| J2R | -4.81 | < 0.0001 |
| CR | -5.44 | < 0.0001 |
| Tipping point |  |  |
| Delta = 0 mmHg | -5.88 | < 0.0001 |
| Delta = 2 mmHg | -5.58 | < 0.0001 |
| Delta = 4 mmHg | -5.28 | < 0.0001 |
| Delta = 6 mmHg | -4.98 | < 0.0001 |
| Delta = 8 mmHg | -4.68 | < 0.0001 |
| Delta = 10 mmHg | -4.38 | < 0.0001 |

CR = copy-reference; DB-WD = double-blind withdrawal: J2R = jump-to-reference; LS mean = least squares mean; mFAS = modified Full analysis set; SiSBP = sitting systolic blood pressure; uAOBPM = unattended automated office blood pressure measurement.

**Table S3. Overview of additional sensitivity analyses DB-WD part – difference to placebo for changes in SiSBP (mmHg) from DB-WD baseline to Week 40 (uAOBPM), modified Full analysis set**

|  | **Aprocitentan 25 mg** | |
| --- | --- | --- |
| **Analysis** | **LS Mean** | **p-value** |
| Main analysis for SiSBP | -5.82 | < 0.0001 |
| Regulatory agency 1 |  |  |
| Multiple Imputation using retrieved dropout for SiSBP | -5.95 | <0.0001 |
| Baseline Multiple Imputation for SiSBP | -4.96 | <.0001 |
| Implement ITT principle by Multiple imputation under MAR (rejected by agency) | –5.89 | < 0.0001 |
| Implement ITT principle by Multiple imputation using retrieved drop out (after discussion with agency) | **–5.72** | **< 0.0001** |
| Regulatory agency 2 |  |  |
| Multiple imputation (1) analysis | –5.79 | < 0.0001 |
| Multiple imputation (2) analysis | –5.74 | < 0.0001 |
| J2R analysis | –4.81 | < 0.0001 |

The result in bold was eventually included in the Agency’s label.

SiSBP = sitting systolic blood pressure; J2R= Jump to reference
